# Obstructive sleep apnea in patients with laryngeal cancer after supracricoid or vertical partial laryngectomy

**DOI:** 10.1186/s40463-019-0347-6

**Published:** 2019-06-03

**Authors:** Lei Ouyang, Liang Yi, Lin Wang, Qinglai Tang, Xinming Yang, Shisheng Li

**Affiliations:** Department of Otolaryngology, Head and Neck Surgery, The Second Xiangya Hospital, Central South University, Changsha, 410011 Hunan People’s Republic of China

**Keywords:** obstructive sleep apnea, laryngeal carcinoma, supracricoid partial laryngectomy, vertical partial laryngectomy

## Abstract

**Objective:**

To investigate whether partial laryngectomy is a risk factor for obstructive sleep apnea (OSA) and the effect of different partial laryngectomy methods on OSA.

**Method:**

A prospective study was carried out involving 40 patients who underwent supracricoid partial laryngectomy (SCPL) (24) or vertical partial laryngectomy (VPL) (16) for carcinoma of the larynx. Apnea-hypopnea index (AHI) and oxygen saturation determined by polysomnography (PSG), Epworth sleepiness scale (ESS) score, and body mass index (BMI) were evaluated in patients before surgery, on the day of tracheal tube removal and three months later. In patients who developed apnea, laryngoscopy, Muller’s test, computer tomography (CT) and dynamic sleep magnetic resonance imaging (MRI) were performed to assess the location of airway stenosis and collapse.

**Results:**

The AHI (*P*<0.001) increased and the lowest oxygen saturation (*P*<0.001), ESS score (*P*<0.001) and BMI (*P*=0.017) decreased after extubation compared with before surgery. Three months after extubation, the same changes were found in AHI (*P*<0.001) and the lowest oxygen saturation (*P*<0.001), but the ESS score (*P*<0.001) increased compared with that preoperatively. The AHI in the SCPL group was significantly higher than that in the VPL group post-operatively (*P*=0.010), while the miniSpO2 in the SCPL group was lower than that of the VPL group (*P*=0.022). Laryngoscopy showed that the patients with partial excision of the larynx had a narrowed retropalatal and retrolingual space post-operatively. Muller's test showed the collapse of the retropalatal and retrolingual space, and the CT scan showed that the tongue root was positioned lower in the SCPL group. Compared with the retropalatal and retrolingual space in the expiratory phase according to dynamic sleep MRI, the space in the inspiratory phase was clearly decreased.

**Conclusion:**

Laryngeal function preservation surgery for laryngeal cancer results in the occurrence of OSA by altering the anatomical structure of the larynx and pharynx. OSA was more severe in patients undergoing SCPL than in patients undergoing VPL. The effect of partial laryngectomy on OSA may be related to the surgical method used.

## Background

Obstructive sleep apnea (OSA) is a relatively common and severe syndrome that affects 3-4 percent of males and 2 percent of females [1]. Patients with OSA may experience loud snoring, oxygen desaturation, frequent arousals, and disruption of sleep. Increasingly, OSA is also being recognized as an independent risk factor for hypertension, stroke, abnormal glucose metabolism and coronary heart disease [2–4]. Factors that increase the vulnerability for the disorder include age, male sex, obesity, family history, menopause, craniofacial abnormalities, and certain health behaviors, such as cigarette smoking and alcohol use [5]. In addition, a series of studies have shown that abnormality of the upper airway anatomy is one of the major risk factors for OSA [6, 7].

Laryngeal cancer is one of the most common malignant tumors in otolaryngology head and neck surgery. Surgical resection, radiotherapy and chemotherapy are the main treatment methods for laryngeal carcinoma. In the early stages, partial laryngectomy is the first choice of treatment [8, 9]. Supracricoid partial laryngectomy (SCPL) and vertical partial laryngectomy (VPL) have been used in cases of non-advanced laryngeal cancer to preserve some of the larynx’s vital functions [10, 11]. Structural alterations to the upper airways consequent to laryngeal function preservation surgery may predispose individuals to OSA. To date, the incidence of OSA in laryngeal neoplasm patients and, in particular, the relationship with the various types of partial laryngectomy remain unclear and poorly defined. Some case reports and small case series have shown that patients who underwent partial laryngectomy had a high incidence of OSA and that there was no change in the incidence of OSA after surgery using different surgical methods, whereas another study reported that OSA was more severe in patients who underwent VPL than in the individuals who underwent horizontal laryngectomy [12–16].

The identification and treatment of OSA should be a considerable factor in the overall quality of life of laryngeal cancer patients. The study aimed to explore whether surgical treatment of laryngeal cancer is a risk factor for OSA and the effect of different partial laryngectomy methods on OSA.

## Method

### Study Design and Patients

A prospective study was carried out involving 40 patients who underwent SCPL or VPL for squamous cell carcinoma of the larynx at the Second Xiangya Hospital of Central South University between June 2015 and March 2017. The study protocol was approved by the research ethics committee of the Second Xiangya Hospital of Central South University, and written informed consent was obtained from each patient.

Briefly, the inclusion criteria were the following: (1) preoperative or intraoperative diagnosis of laryngeal carcinoma, (2) absence of preoperative laryngeal obstruction and dyspnea, (3) absence of dentofacial abnormality, (4) absence of severe basic lung disease, (5) no history of sedative and hypnotic drugs, (6) postoperative smooth tracheostomy tube removal, (7) absence of postoperative radiotherapy; and (8) lack of preoperative severe OSA (AHI > 30).

The clinical staging was defined according to the Tumor, Node, Metastasis (TNM) classification system of the International Union Against Cancer staging system (7th edition, 2010). Body mass index (BMI) is calibrated based on World Health Organization (WHO) standards.

The choice of operation and surgical steps are described in previous studies [10, 17–19]. All patients with open surgery were given regular dressing changes and training on swallowing function during hospitalization. Additionally, the gastric tube was removed before hospital discharge, and the patients were transitioned to a half fluid diet and gradually to a normal diet. Telephone follow-up and regular review were performed in all patients who had undergone surgical treatment. The patients should be examined by fiberoptic laryngoscopy during the outpatient review. If there is no recurrence of the tumor or airway stenosis, the tracheal tube was removed and the fistula was sutured.

### Polysomnography

All the patients were sent to the sleep lab to undergo polysomnography (PSG) within a week begore operation according to routine protocols [20]. Respiratory events and the AHI were staged based on the American Academy of Sleep Medicine (AASM) standardization [21, 22] . The test was used to assess the AHI and minimum oxyhemoglobin saturation. OSA severity is defined as severe for AHI > 30, moderate for AHI ≤ 30 and ≥ 15, and mild for AHI <15 and ≥ 5.

### Epworth sleepiness scale

Enrolled patients were evaluated with the ESS to assess the subjective degree of daytime sleepiness [23]. Mild sleepiness was defined as an ESS score ≤12, moderate sleepiness as an ESS score between 13 and 17, and severe sleepiness as an ESS score from 18 to 24[24].

### Flexible pharyngoscopy with Müller's maneuver

Flexible pharyngoscopy with Müller's maneuver (FPMM) was carried out to examine the subjects’ upper airway using an electronic laryngoscope. It was used to evaluate airway deformity or the anatomical structure of the stenosis of the nasal cavity, nasopharynx, oropharynx, hypopharynx and larynx before the surgery, with emphasis placed on the levels of obstruction. The uvula was used as the anatomical landmark at the retropalatal level, and the tip of the epiglottis was used as the landmark at the retrolingual level. Measurements were taken at the end of quiet respiration, during Mueller’s maneuver, and in the supine position at two levels [25]. Three months after removal of the tracheal tube, the airway condition of the patients was evaluated again, and the narrowest site was observed. A video recording of the entire examination was made, which included quiet respiration and Mueller’s maneuver in the supine position. The changes in the airway structure were compared with the preoperative condition.

### Computed tomography

An upper airway CT scan was performed in all patients before the operation and as needed for the surgery. After the operation, the patients with OSA underwent CT again. The changes in the retropalatal and retrolingual spaces were measured.

### Sleep dynamic magnetic resonance imaging

All the subjects were scanned after 10 p.m. The MRI results were assessed by a radiologist and an otolaryngologist. Subjects were supine in the orbito-auricular plane at a 90-degree angle to the horizontal plane and wore earplugs to avoid noise interference. Pulse rate, oxygen saturation and snoring sounds were continuously recorded during the scan. The specific technical parameters were set according to the report [26].

### Statistical analysis

The results are expressed as means ± SD. Quantitative data were analyzed by a paired Student’s t test for two-sided hypothesis. To compare the SCPL and VPL groups, we used two-way repeated measures multivariate ANOVA. In all cases, P < 0.05 was considered statistically significant, and all analyses were performed with SPSS 19.0 (SPSS, IBM, Armonk, NY).

## Results

There were 8 non-smokers and 32 smokers, as well as 6 non-drinkers and 34 drinkers. Their ages ranged from 44 to 67 years. Among the 40 patients, 24 underwent SCPL and 16 underwent VPL. Of the 40 cases, 17 patients had no OSA (AHI<5) before the operation. Three months after extubation, 33 patients had OSA, 2 of whom had severe OSA. The clinical characteristics of the patients are listed in Table 1. The details regarding AHI, the lowest oxygen saturation, ESS score and BMI are presented in Table 2.

**Table 1:** Clinical characteristics of the patients undergoing laryngeal functional preservation surgery

Details	N
Patients	40
Age (years)	
51 – 60	22
> 60	18
Gender	
Male	37
Female	3
Mini SaO2(%)	
≥90%	20
≥85%, <90%	17
≥65%, <85%	3
< 65%	0
AHI	
<5	17
<15, ≥5	21
≤30, ≥15	2
ESS scores	
≤6	26
≤11, >6	14
≤16, >11	0
>16	0
BMI (kg/m2)	
<18.5	1
<25, ≥18.5	32
<28, ≥25	7
<32, ≥28	0
TNM classification	
T1N0M0	20
T2N0M0	15
T3N0M0	5

*AHI* apnea/hypopnea index, *ESS* epworth sleepiness scale, *BMI* body mass index

**Table 2:** Polysomnography, epworth sleeping scale, anthropometric, and surgical medthod of the patients

Patient	AHI			Mini SaO_2_			Epworth			BMI			Surgical Method
	PRE	TR	PO	PRE	TR	PO	PRE	TR	PO	PRE	TR	PO	
1	8.5	9.7	9.1	88	85	84	3	3	3	64	63	65	SCPL
2	4.4	16.1	20.2	90	84	78	8	5	11	57	58	64	SCPL
3	6.9	0.7	8.9	96	94	82	9	7	10	73	63.3	71.5	SCPL
4	2.2	7.3	6.9	87	82	83	10	7	7	66	54	62	SCPL
5	1.2	2.4	3	87	87	86	5	4	7	52.2	53.7	54	SCPL
6	0.4	15	16.7	95	78	80	5	5	12	57	53	55	SCPL
7	8.6	21.9	23.5	86	78	72	2	2	7	61	57	61	SCPL
8	1.8	0.1	2.8	82	89	86	8	5	7	57	63	60	VPL
9	1.6	3.8	2.3	92	86	88	4	3	4	55	48	50	VPL
10	4	17.2	13.4	85	82	68	3	3	10	73	65	70	SCPL
11	6.2	15.7	17.7	90	80	78	1	1	8	70	62	65	SCPL
12	2.4	7.1	3.5	88	85	81	4	3	7	52.4	58	50	VPL
13	3.1	2.8	6.8	87	86	85	6	3	5	59	61.8	64	SCPL
14	0.8	6.9	4.3	94	87	83	3	5	4	50	54	53.5	SCPL
15	7.2	18.5	21.5	86	78	76	8	4	11	67	72.7	75	VPL
16	6.5	5.1	7.7	88	86	86	3	3	5	60	55	58	VPL
17	8.3	6.7	10.2	90	88	82	5	4	5	63	62	60	VPL
18	15.7	17.5	20.2	88	86	83	9	4	10	69	70	72	VPL
19	10.7	13.4	15.1	86	85	84	9	5	9	70	66	68	SCPL
20	4.7	19.1	22.3	95	90	84	5	4	8	55	56	53	SCPL
21	5.2	3.2	6.7	92	91	88	4	4	4	64	65	68	SCPL
22	8.2	9.9	11.2	88	84	80	6	4	7	68	64	62	VPL
23	17.9	21.5	25.7	84	82	78	10	6	13	69	70	72	SCPL
24	5.6	19.9	23.6	92	89	85	6	6	12	67	63	65	SCPL
25	6.2	7.7	8.5	90	89	91	6	5	5	60	57	61	VPL
26	4.5	22.1	24.9	96	85	75	5	4	11	62	60	65	SCPL
27	7.8	9.1	8.8	94	92	90	6	4	5	61	58	67	VPL
28	12.7	28.1	30.5	91	78	72	10	6	14	64	65	70	SCPL
29	8.4	13.4	17.9	87	85	82	8	6	9	67	64	63	VPL
30	2.9	8.2	12.8	92	88	84	8	7	11	57	58.5	60	VPL
31	12	25.7	27.4	85	78	75	7	6	13	57	60	62	SCPL
32	4.2	20.8	26.5	90	82	72	2	4	12	70	73	75	SCPL
33	3.4	6.9	4.5	90	92	89	4	5	4	59	56	57	VPL
34	9.6	8.7	11.2	88	85	80	5	5	7	60	55	56	VPL
35	5.8	3.8	7.1	93	88	87	4	6	5	56	55	58	VPL
36	14.1	18.5	20.2	85	83	78	8	6	9	67	68	70	SCPL
37	2.6	5.5	4.3	92	95	90	3	4	3	66	62	68	VPL
38	11.2	15.9	18.4	83	87	81	7	5	8	57	53	55	SCPL
39	10.4	26.6	33.7	88	82	64	5	6	15	63	60	65	SCPL
40	4.9	9.9	13.7	90	86	89	6	5	8	57	53	54	SCPL

*AHI* apnea/hypopnea index, *BMI* body mass index, *PRE* preoperation, *TR* night of tube removal, *PO* 3 months after tube removal, *SCPL* supracricoid partial laryngectomy; *VPL* vertical partial laryngectomy.

**Fig 1:**
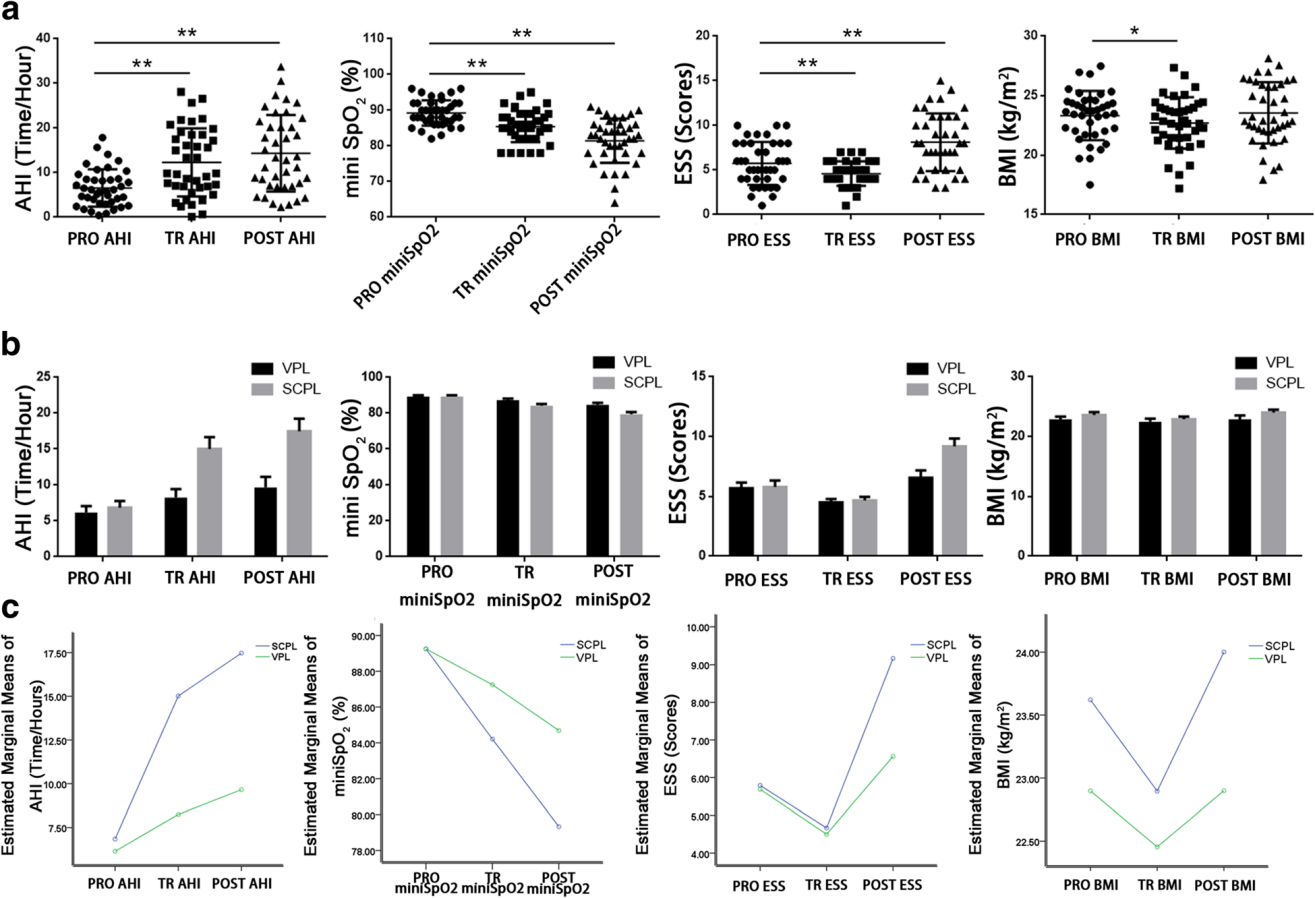
Statistical analysis of AHI, mini SpO_2_ value, ESS scores and BMI of the 40 patients before surgery, at the time of extubation, and 3 months after extubation (**a**). Descriptive statistics of AHI, mini SpO_2_ value, ESS scores and BMI of patients undergoing SCPL or VPL in 3 time points (**b**). Estimated marginal means of AHI, mini SpO_2_, ESS and BMI with two groups (**c**). *AHI, apnea/hypopnea index; ESS, epworth sleepiness scale; BMI, body mass index; PRE, preoperation; TR, night of tube removal; PO, 3 months after tube removal; SCPL, supracricoid partial laryngectomy; VPL, vertical partial laryngectomy.* *P < 0.05, **P < 0.01

The AHIs (times/hours) of the 40 patients before surgery, at the time of extubation, and 3 months after extubation were 6.57±4.19, 12.31±7.64, and 14.34±8.59; the minimum oxygen saturation values (%) were 89.25±3.57, 85.43±4.37, and 81.47±6.22; the ESS scores were 5.75±2.42, 4.60±1.37, and 8.13±3.24; and the BMIs (kg/m^2^) were 23.33±2.07, 22.72±2.17, and 23.56±2.57, respectively. Compared with pre-surgery values, AHI (*t*=-5.661, *P*<0.001) increased, and the lowest oxygen saturation (*t*=5.431, *P*<0.001), ESS score (*t*=4.128, *P*<0.001) and BMI (*t*=2.501, *P*=0.017) decreased at the time of extubation. Three months after extubation, the same changes were found in AHI (*t*=-6.980, *P*<0.001) and the lowest oxygen saturation (*t*=7.763, *P*<0.001), but the ESS scores (*t*=-4.935, *P*<0.001) increased compared with the preoperative scores. With regard to BMI, we found that there was no significant change between the preoperative and postoperative values (*t*=-1.032, *P*=0.308) (Fig. 1a).

To further analyze the effect of different partial laryngectomy on postoperative sleep respiration, the patients were divided into the supracricoid partial laryngectomy group and vertical partial laryngectomy group according to operation mode. The results showed that the AHI of the SCPL group at the time of extubation was 15.01±8.04, which was higher than the preoperative value (6.85±4.54, *t*=-5.864, *P*<0.001). Moreover, their miniSpO2 and ESS scores both declined (miniSpO2: 84.21±4.31, *t*=5.346, *P*<0.001, ESS score: 4.67±1.52, *t*=3.145, *P*<0.001) compared with before surgery (miniSpO2: 89.25±3.96, ESS score: 5.79±2.70). Three months after extubation, AHI and ESS scores were significantly increased compared with the preoperative values (AHI: 17.47±8.73, *t*=-7.196, *P*=0.002, ESS score: 9.17±3.28, *t*=-4.811, *P*<0.001), while the miniSpO2 value decreased (miniSpO2: 79.33±6.43, *t*=7.110, *P*<0.001). Interestingly, we found the same trend in the VPL group, which had AHI values of 6.15±3.70, 8.25±4.83 and 9.66±6.01; miniSpO2 values of 89.25±3.02, 87.25±3.91 and 84.69±4.36; and ESS scores of 5.69±2.00, 4.50±1.16 and 6.56±2.53 before surgery, at the time of extubation and 3 months after extubation, respectively, and the differences were significant (Fig. 1b).

Comparing the data between the two groups, we found that the AHI in the SCPL group was significantly higher than that in the VPL group postoperatively (*F*=7.420, *P*=0.010), while the miniSpO2 value was lower than that in the VPL group (*F*=5.684, *P* =0.022). With regard to the ESS score, although surgery had an impact on the score, no difference was found between different surgical treatments (*F*=2.454, *P* =0.126). The two groups of patients did not show any difference in BMI (*F*=1.215, *P* =0.277) (Fig. 1c).

**Fig 2:**
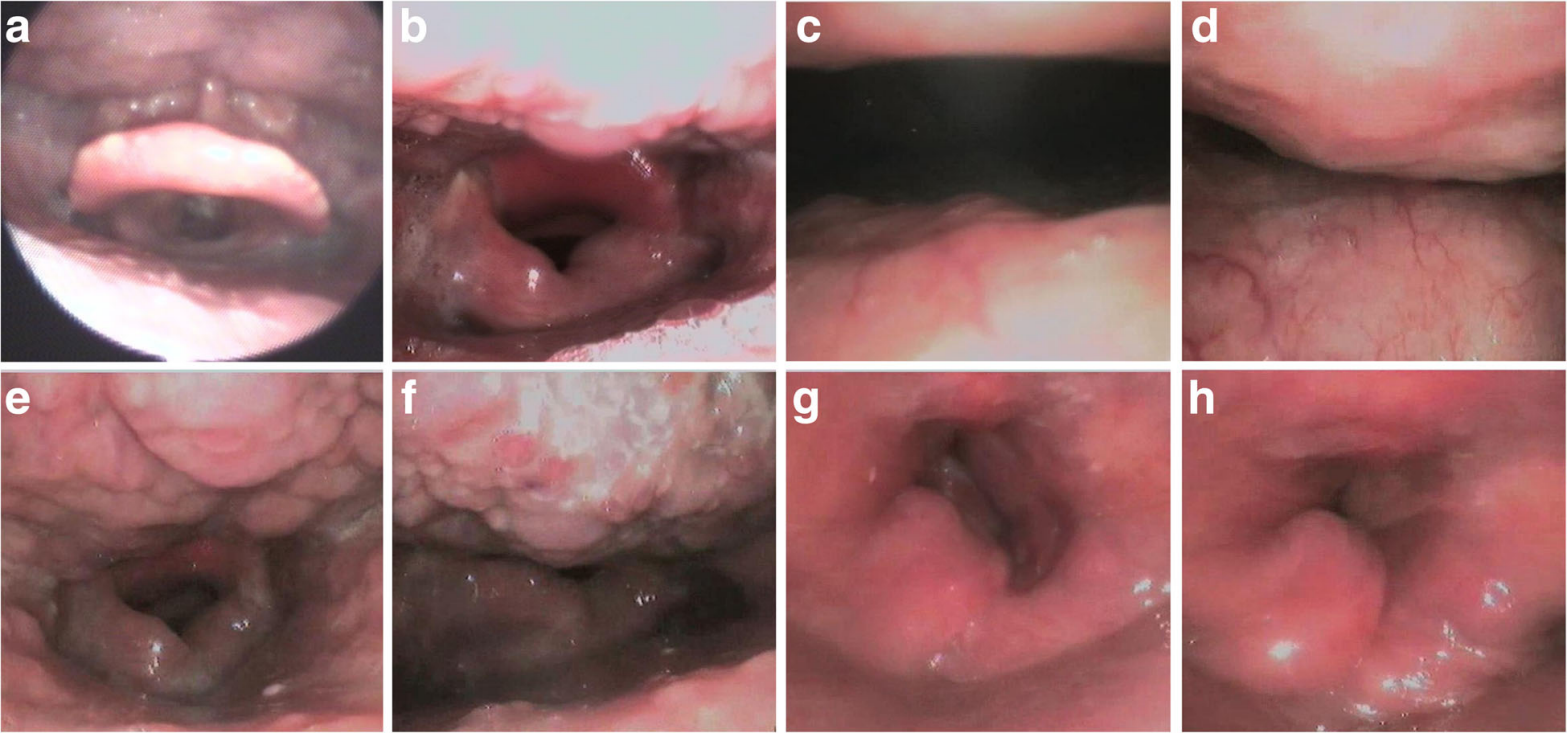
Retrolingual space of one patient undergoing SCPL before the surgery (**a**) and 3 month after extubation (**b**). Retropalatal (**c**) and retrolingual (**e**) space during the end of quiet respiration; Retropalatal (**d**) and retrolingual (**f**) space during Mueller’s maneuver with one patient undergoing SCPL. Retrolingual space in the expiratory phase (**g**) and aspirated phase (**h**) with one patient undergoing VPL

Further examination was performed in 18 patients with moderate OSA (AHI 3 months after extubation > 15). Electronic laryngoscopy showed that the retrolingual space in 15 VPL group patients was significantly narrower than that preoperatively (Fig. 2a and b ). We observed through Müller's maneuver that the retropalatal and retrolingual spaces collapsed conspicuously during inspiration, and there was substantial mucosal hyperplasia around the entrance of the airway at the epiglottis level (Fig. 2c, d, e,and f). However, we found no significant changes in the retropalatal and retrolingual spaces in 3 patients in the VPL group, while mucosal hyperplasia was still seen and moved partially inward during inspiration (Fig. 2g and h).

**Fig 3.**
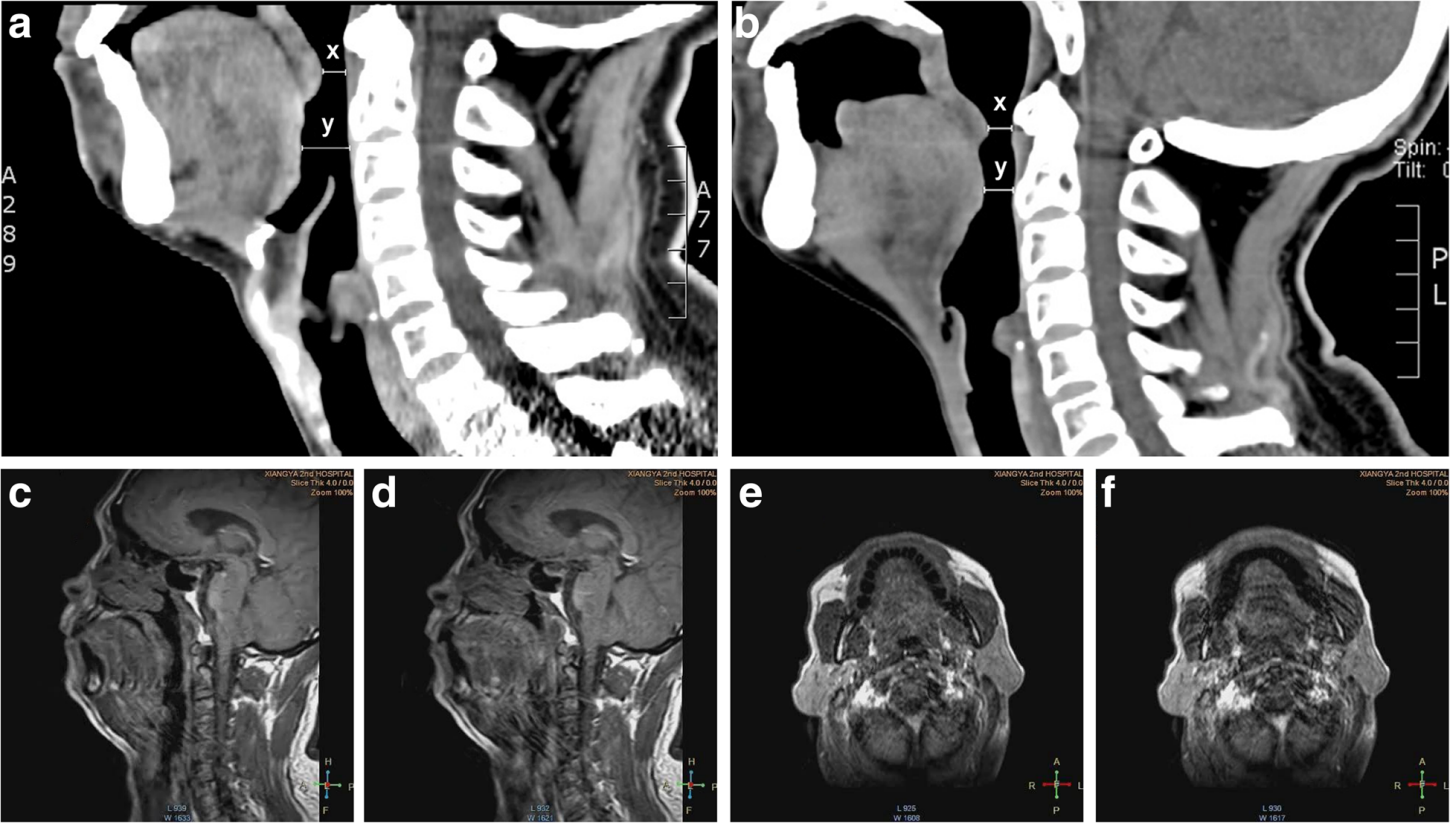
Anteroposterior diameter of retrolingual space (x) and retropalatal space (y) in CT sagittal plane of one patient undergoing SCPL before the surgery (**a**) and postoperation (**b**). Retropalatal and retrolingual space in the expiratory phase (**c**) and aspirated phase (**d**) in sagittal plane of dynamic sleep MRI; Retropalatal and retrolingual space in the expiratory phase (**e**) and aspirated phase (**f**) with one patient undergoing SCPL in transverse plane of MRI

Moreover, in the postoperative CT images of 15 VPL group patients, we observed a clear change in the tongue position in the sagittal plane. The anteroposterior diameter of the retrolingual space (x=7.00±0.82 mm) was clearly smaller than that before surgery (preoperative x=11.00±4.08 mm, *P*=0.001); similarly, the anteroposterior diameter of the retropalatal space (y=4.75±1.26 mm) was also decreased (preoperative y=5.50±1.29 mm, *P* =0.003) (Fig. 3a and b). Upon examination of the 18 patients by dynamic sleep MRI, we found that compared with the retropalatal and retrolingual space in the expiratory phase, the space in the inspiratory phase was clearly decreased (Fig. 3c, d, e, and f).

## Discussion

The treatment of head and neck cancer has evolved over the past century. The main changes over the past 20 years have not been related to treatment or reconstruction but rather to the quality of life of patients. Tumor resection that results in a poor quality of life is not an ideal outcome of modern treatment. The identification and treatment of OSA may be an important factor in improving quality of life. The effects of head and neck tumors, especially laryngectomy, on sleep-disordered breathing are rarely reported. We observed the changes in sleep breathing after laryngeal function preservation surgery in 43 patients with laryngeal carcinoma associated with the changes in the anatomical structure of the pharynx and larynx. Compared with the values before surgery, the AHI and miniSpO2 values both changed after partial laryngectomy, which indicated that sleep-disordered breathing is aggravated and that partial laryngectomy may promote the occurrence or aggravation of OSA. However, patients' ESS scores at the time of extubation were lower than those preoperatively. The ESS score represents the daytime drowsiness of patients and reflects a patient's lethargy in the past month. All the patients breathed with a tube from after surgery to the time of extubation, and some of them had already been diagnosed with mild OSA before the surgery. Tracheotomy is the most effective surgical treatment for OSA patients, especially for those with severe OSA. Therefore, the ESS score at the time of extubation reflected the drowsiness of patients after tracheotomy. The current view is that weight gain is a risk factor for OSA [27]. In this study, the BMI of the patients postoperatively was slightly lower than that before surgery. The diagnosis of cancer, effects of surgery on the diet, and mental and psychological factors of the patients may have resulted in the weight loss. To advance our study, we observed the effect of laryngeal cancer surgery on later sleep and quality of life. The assessment of sleep breathing 3 months after extubation showed that the AHI continued to increase while the miniSpO2 value was further reduced, indicating that surgical operation aggravated sleep-disordered breathing. The ESS score 3 months after extubation was consistent with the AHI, revealing that severe anoxia appeared in the 3 months after the removal of the tracheal cannula due to upper airway stenosis. The BMI did not differ between the presurgical value and the value 3 months after tracheal tube removal, so weight changes did not affect sleep breathing. A study of 24 patients undergoing head and neck tumor surgery confirmed that surgery was a risk factor for OSA, which was consistent with our findings in laryngeal cancer [28].

The anatomic etiology of OSA patients is important for current Sleep Medicine, as laryngeal and pharyngeal structures are significantly altered by laryngeal function conservation surgery. During laryngoscopy for patients with clear postoperative sleep-disordered breathing, we found that compensatory repair and proliferation of mucosa appeared around the entrance of the airway at the arytenoid cartilage in both the VPL and SCPL groups. This pharyngomalacia accumulated at the inlet of the airway, which moved inward during inspiration. This movement resulted in stenosis of the airway inlet, which may lead to the occurrence of OSA. The thyroid cartilage of the bony airway could support the cavum laryngis when inhaling, avoiding collapse and maintaining the function of sound. All the thyroid cartilage was removed in the SCPL group, while a unilateral cut was performed in the VPL group. The thyroid cartilage that supports the laryngeal cavity was replaced by soft tissue. However, soft tissue in the airway provides no support for the laryngeal cavity, such that during inhalation, this section of the airway collapsed more easily than preoperatively. Muscle tension of the upper airway was necessary to maintain the upper airway patency, and upper airway hypotonia during sleep could lead to OSA [29]. Partial laryngectomy damaged the supporting structures and architecture of the hypopharynx and larynx. The scar tissue that remained after healing did not have the same tension as that of the original neuromuscular tissue. The soft tissues of the lower pharynx and larynx were more prone to collapse during sleep and led to the occurrence of OSA.

According to the lesion location and extent of invasion, different surgical methods could be selected for laryngeal function preservation operations [30]. The different resection and reconstruction methods result in different changes in pharynxstructure. SCPL and VPL were the most commonly used procedures at the time of the study. In this study, we divided the patients into two groups according to the surgical method to thoroughly investigate the effects of different surgical methods on sleep and respiratory disorders. The results showed that the pattern of change in the AHI, miniSpO2 and ESS score was the same in both the SCPL and VPL groups at the three time points (before surgery, at the time of extubation and 3 months after extubation). The AHI of the SCPL group was much higher than that of the VPL group after the operation, while the miniSpO2 value was clearly lower. Although no difference in the ESS score and BMI was found between the two groups, the changes in sleep apnea in the SCPL group were more significant than those in the VPL group. The results showed that patients undergoing SCPL were more likely to have more severe OSA than those undergoing VPL. An evidence showed there was no difference in the incidence of OSA after the SCPL and VPL operations between the two groups in 22 patients [13]. Interestingly, another study involving 14 people indicated that OSA was more severe in patients offered vertical laryngectomy than in the individuals subjected to horizontal laryngectomy [16]. Those results were inconsistent with our findings, which may be related to a retrospective study with a small series. These two studies failed to assess the preoperative sleep-disordered breathing status of patients and only assessed this status in the patients after the operation; furthermore, they did not explore the causes and mechanisms of this phenomenon. To clarify the clinical mechanism of increased sleep-disordered breathing in laryngeal cancer patients after laryngectomy, we compared the anatomical structure of the cavum pharyngis before the surgery and three months after extubation by electronic laryngoscopy, upper airway CT and dynamic MRI. The cross-sectional area of the retropalatal space is related to the severity of OSA [31]. The retropalatal and retrolingual spaces in the SCPL group decreased significantly; however, no significant changes were observed in patients with typical OSA in the VPL group. The hyoid bone plays an important role in keeping the upper airway patency; the hyoid bone at the level of the mental tubercle can cause the genioglossus to pull the tongue forward, and the lower hyoid can lower the position of the tongue and lead to stricture of the posterior lingual airway space [6]. The posterior lingual space is directly related to the severity of OSA [32]. After resection of the thyroid cartilage, the epiglottis or hyoid bone directly anastomosed with the cricoid cartilage, and the hyoid bone moved down, driving the base of the tongue backward. The anteroposterior diameter of the posterior airway space (PAS) is the anteroposterior diameter of the narrowest area of the retrolingual space and was negatively correlated with the severity of OSA [32]. In the SCPL group, we observed that the tongue was shifted back and down by CT. The anteroposterior diameter of the PAS decreased significantly with the increased severity of OSA. These findings illustrate that the decrease in the PAS after the operation of laryngeal cancer is an important pathogenic factor of OSA. Moreover, the tongue pushed the soft palate backward, resulting in less retropalatal space. These changes were more pronounced during sleep based on dynamic sleep MRI.

Recent research has focused more on the quality of life of patients, instead of the survival rate and relapse-free rate, in the treatment of malignant tumors. Due to the position and effects on function of head and neck cancers, surgeons also need to consider aspects such as physical appearance, function (voice, swallowing), and quality of life. Long-term sleep-disordered breathing not only seriously affects the quality of life of patients but may also lead to complications of multiple organ systems, such as hypertension, diabetes, and coronary heart disease [33]. The purpose of partial laryngectomy is to ensure the function of the larynx after complete removal of the tumor and ensure safe incisal margins. Therefore, it is not feasible to prevent OSA after laryngectomy by changing the operation type. For the general population with OSA, continuous positive airway pressure (CPAP) is effective in relieving fatigue and reducing neurocognitive impairment or depression. The more serious the degree of OSA, the better the compliance of patients [34]. However, in patients with OSA after laryngeal partial laryngectomy, OSA is mostly moderate, and compliance with CPAP is not optimal. The cause of OSA after laryngectomy is unique and can be evaluated by CT or laryngoscopy. Different treatments can be used for OSA associated with different causes. For example, for stenosis at the epiglottis level caused by hyperplasia of the laryngeal mucosa, laser therapies can be used to excise excess mucosa and improve airway stenosis. However, OSA caused by narrowing of the retrolingual space can be treated with coblation channeling at the tongue base, CPAP and oral appliances. Of course, multiple level surgery, such as palate surgery with tongue base surgery, might achieve better effect. In addition, after laryngeal cancer surgery, under the premise of ensuring nutrition, it is recommended that patients should properly control their weight to reduce fat accumulation in the neck after laryngeal cancer surgery to reduce the occurrence of OSA.

This study showed that laryngeal cancer patients had laryngeal structure changes that caused the occurrence of OSA. SCPL is more likely to aggravate sleep-disordered breathing than VPL. The study results demonstrate that patients who undergo surgery for laryngeal function preservation, especially SCPL, should be followed up to assess their sleep condition and quality of life. For patients with moderate or severe OSA, early intervention should be attempted.

## Conclusion

We observed that laryngeal function preservation surgery for laryngeal cancer results in the occurrence of OSA by altering the anatomical structure of the larynx and pharynx. OSA was more severe in patients undergoing SCPL than in patients undergoing VPL. The effect of partial laryngectomy on OSA may be related to the surgical method used.

## Data Availability

The datasets used and analysed during the current study are available from the corresponding author on reasonable request.

## Abbreviations

AASM: American Academy of Sleep Medicine
AHI: Apnea-hypopnea index
BMI: Body mass index
CPAP: Continuous positive airway pressure
CT: Computer tomography
ESS: Epworth sleepiness scale
FPMM: Flexible pharyngoscopy with Müller's maneuver
MRI: Magnetic resonance imaging
OSA: Obstructive sleep apnea
PAS: Posterior airway space
PSG: Polysomnography
SCPL: Supracricoid partial laryngectomy
TNM: Tumor, Node, Metastasis
VPL: Vertical partial laryngectomy
WHO: World Health Organization
